# Our Next Issue

**Published:** 1883-11-20

**Authors:** 


### *OUR NEXT ISSUE.*

Our December number will contain the Index of the present or 13th volume of THE RECORD. Few people are aware of the immense labor and expense required in preparing and publishing an Index. We are in the habit of making a very full one, and so numerous are the items in our Journal, resulting from its condensed and varied matter, that the subscriber who binds his volume at the end of the year, can find something practical and interesting upon almost any subject concerning which information may be desired.

